# Investigation of the Substrate Rotation Speed Effect on the Morphology and Tribo-Mechanical Behavior of CrAlN Thin Films Using DC Magnetron Sputtering

**DOI:** 10.3390/ma19153167

**Published:** 2026-07-24

**Authors:** Khalil Aouadi, Aurélien Besnard, Corinne Nouveau, Alex Montagne, Yahya Agzenai Ben Salem

**Affiliations:** 1 Engineering and Durability of Materials, Department of Materials Science, College of Chemical Sciences and Engineering (CCSE), Mohammed VI Polytechnic University (UM6P), Benguerir 43150, Morocco; 2 Applied Mechanics and Systems Research Laboratory (LR03ES06), Tunisia Polytechnic School, University of Carthage, BP 743, Rue El Khawarizmi, La Marsa 2078, Tunisia; 3 Université Marie et Louis Pasteur, SUPMICROTECH, CNRS, Institut FEMTO-ST, F-25000 Besançon, France; aurelien.besnard@femto-st.fr; 4 Arts et Métiers Institute of Technology, LABOMAP, HESAM Université, UBFC, F-71250 Cluny, France; corinne.nouveau@ensam.eu; 5 LAMIH, Université Polytechnique Hauts-de-France, UMR 8201, F-59313 Valenciennes, France; alex.montagne@uphf.fr; 6 High Throughput Multidisciplinary Research Laboratory (HTMR), College of Chemical Sciences and Engineering (CCSE), Mohammed VI Polytechnic University (UM6P), Ben Guerir 43150, Morocco; yahya.agzenaibensalem@um6p.ma

**Keywords:** CrAlN thin films, substrate rotation speed, morphology, hardness, wear resistance

## Abstract

**Highlights:**

- CrAlN thin films with different substrate rotation speed were deposited via DC magnetron sputtering.
- The increase in substrate rotation speed did not affect CrAlN coating microstructure.
- Increasing the substrate rotation speed in a dual-axis planetary PVD configuration enhanced CrAlN coating hardness by reducing shadowing effects and promoting denser microstructure.
- Superior wear resistance was observed with increasing substrate rotation speed. However, this parameter did not affect the coefficient of friction.

**Abstract:**

In this work, CrAlN thin films were used to improve the lifespan of cutting tools. The CrAlN thin films were deposited on X50CrMoV8 substrates and silicon using DC reactive magnetron sputtering. The influence of substrate rotation speed (varied between 0.5 and 3 rpm) on the morphology, mechanical properties, and tribological properties of the thin films was investigated. The results showed that this process parameter had no significant influence on chemical composition or morphology. Nevertheless, the mechanical properties were enhanced. Specifically, CrAlN hardness increased significantly with the rise in substrate rotation speed from 0.5 to 1.5 rpm. Additionally, the adhesion strength of CrAlN coatings followed the same trend as hardness. When the substrate rotation speed increased, the residual stress shifted from tensile to compressive. Changing the rotation speed did not affect the friction coefficient value but did impact wear resistance. Thin films deposited at 0.5 and 1 rpm exhibited the lowest wear resistance. Once the rotation speed increased, wear behavior improved significantly. The CrAlN thin film deposited at a substrate rotation speed of 1.5 rpm showed the best wear resistance along with the highest hardness and adhesion strength.

## 1. Introduction

During machining, numerous interactions between cutting tools and workpiece components can lead to tool damage, thereby reducing their service life. Several mechanisms contribute to this damage, including wear, plastic deformation, fatigue, and corrosion [1,2,3,4]. Therefore, it is necessary to protect tools with a coating that offers excellent resistance to wear and corrosion. Aluminum-containing nitride coatings, such as CrAlN and TiAlN, are valuable protective materials due to their good wear and oxidation resistance, corrosion resistance, mechanical properties, and thermal stability [5,6,7,8,9]. TiAlN films are widely used to protect tools because of their high hardness [10], good wear resistance [11], and good corrosion resistance [12]. However, TiAlN coatings have a relatively low oxidation resistance and thermal stability beyond 800 °C, which limits their application at high temperatures [13,14]. CrAlN coatings are widely used to protect tools owing to their good wear and oxidation resistance and their good mechanical properties, which can reach 1100 °C [15,16,17]. This excellent oxidation behavior is attributed to the formation of a dense and protective (Al, Cr)_2_O_3_ layer [16,18,19].

CrAlN coatings exhibit exceptional oxidation resistance and thermal stability, making them a focal point of extensive research aimed at their development and optimization. Numerous studies have investigated the impact of deposition parameters in physical vapor deposition (PVD) techniques to enhance the mechanical and tribological properties of CrAlN coatings as well as their adhesion to metallic substrates. Yu et al. [20] deposited CrAlN coatings using a modified ion beam-enhanced magnetron sputtering system. They studied the effect of substrate bias voltage and the Al/(Al + Cr) ratio on the properties of these coatings. The results showed that at a bias voltage of −120 V and an Al/(Al + Cr) ratio of 40%, the hardness reached its maximum value of 33.8 GPa, and the adhesion was excellent. Also, Ding et al. [21] developed CrAlN films by reactive unbalanced magnetron sputtering and studied the influence of the Al/Cr atomic ratio. They compared the properties of CrN and CrAlN and observed that the hardness of CrN was about 18 GPa, while that of CrAlN reached 30 GPa. They revealed that the wear resistance of CrAlN is approximately six times better than that of CrN film. Ding et al. [22] compared the corrosion resistance of CrAlN and TiAlN films. The coatings were deposited using a vacuum arc reactive deposition process. The results showed that CrAlN exhibited better corrosion resistance than TiAlN coating. Lin et al. [19] proved the good oxidation behavior of CrAlN coatings. They investigated the effect of temperature in the properties of CrN and CrAlN coatings. They noticed that the CrN films oxidized after 600 °C and the CrAlN film oxidized after 1000 °C. Aihu et al. [11] compared properties of TiN, TiAlN, AlTiN and CrAlN coatings deposited by the cathodic arc-evaporation technique. They confirmed that CrAlN exhibits the best anti-spalling and anti-adhesion properties. Khamseh et al. [23] prepared CrAlN films with a pulsed DC reactive balanced magnetron sputtering system. They investigated the influence of the total gas pressure (N_2_/Ar). Results proved that the increase in the sputtering pressure decreased the thin films’ internal stress. Also, they observed that the plastic hardness was high when the total gas pressure was low. Other parameters have been studied to improve the properties of CrAlN coatings. Lv et al. [24] deposited CrAlN thin films using a mid-frequency unbalanced magnetron sputtering system. The effect of the duty cycle on the coatings’ properties was investigated. They revealed that surface roughness, grain size, and the concentrations of Al and N decrease with increasing duty cycle. Increasing this parameter leads to a change in cross-sectional morphology from a random porous structure to a fine continuous columnar structure. It also results in a transition of residual stress from tensile to compressive. The hardness improved due to the denser structure. Other researchers have proposed studying the effect of the process on the properties of CrAlN films. Nouveau et al. [25] examined the properties of CrAlN films obtained by magnetron sputtering using either one target (CrAl) or two separate targets (Cr and Al). They varied the working pressure, bias voltage, and deposition time. The results revealed that films deposited with two targets exhibited better tribological properties, whereas the coating deposited with a single target presented a lower friction coefficient. They attributed this result to the high hardness and Young’s modulus of this coating, which reached 36 GPa. Cao et al. [26] varied nitrogen pressure from 0.04 to 0.07 Pa and studied its impact on the properties of CrAlN coatings. They showed that hardness increases significantly with rising nitrogen pressure, which they attributed to grain refinement and the formation of a solid CrN phase. They also observed that adhesion strength increases with nitrogen pressure up to a point and then decreases, which they ascribed to an increase in internal stress. The CrAlN thin film deposited at 0.05 Pa exhibited the best corrosion resistance owing to the dominant AlN phase structure and its high compactness. He et al. [27] also studied the effect of nitrogen pressure on CrAlN coatings deposited by pulsed laser deposition. They observed that nitrogen pressure influences both the mechanical and microstructural properties.

All the reviewed studies have investigated the effect of input parameters such as nitrogen flow rate, aluminum percentage, and working pressure. However, although some research has explored the influence of substrate rotation on the morphology or chemical composition of thin films, few studies have systematically focused on its simultaneous impact on the morphological, mechanical, and tribological properties of pure CrAlN films. This gap in the literature presents an opportunity to deepen our understanding of the mechanisms linking deposition dynamics to coating performance.

In the present work, we studied a parameter process: the substrate rotation speed. This parameter is a crucial factor in the deposition process affecting the properties of CrAlN thin films. The CrAlN films were synthesized using reactive DC magnetron sputtering on (100) silicon and stainless-steel substrates (X50CrMoV8) with rotation speeds varying between 0.5 and 3 rpm. The main purpose of this study was to investigate the influence of substrate rotation speed on the microstructure, mechanical properties, and tribological properties.

## 2. Materials and Methods

### 2.1. Coatings Deposition

**Figure 1:**
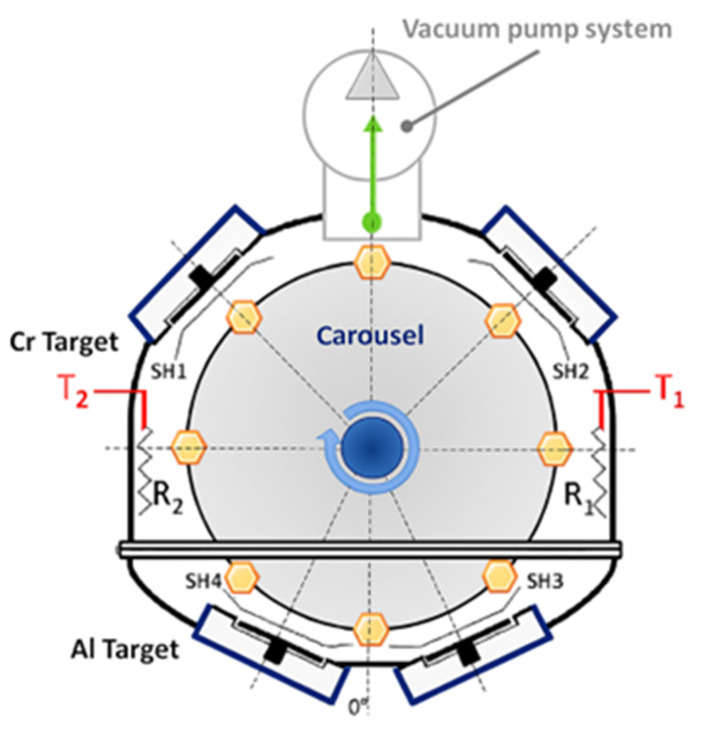
Schematic diagram of the deposition process of CrAlN films.

The CrAlN coatings were deposited on mirror polished silicon (100) with an area of 10 × 10 mm^2^ and a thickness of 380 µm and a stainless-steel sample (X50CrMoV8) (20 × 20 mm^2^ and 5 mm thick) using a direct current reactive magnetron sputtering (KENOSISTEC-KS40V). This industrial deposition system is equipped with four targets, which are installed pairwise. Before deposition, substrates were ultrasonically cleaned in acetone and ethanol for 5 min and then dried under compressed air. Prior to the deposition, the vacuum chamber was evacuated to a pressure less than 2 × 10^−5^ Pa. After this, all substrates were etched in situ by an argon plasma under a bias voltage of −700 V for 10 min to remove surface contamination and to ensure adhesion. During deposition, two targets of Cr and Al with purity of 99.95% were employed. The Cr and targets’ power was set, respectively, at 1500 W and 1000 W. The CrAlN films were realized by rotating the carousel with a two-fold rotation. In this planetary configuration, the substrate holder rotates about its own axis while orbiting the central target axis, ensuring homogeneous exposure to the plasma flux. Figure 1 shows the schematic representation of the deposition system.

The argon and nitrogen flow rates were accurately fixed at 68.8 and 33.3 sccm, respectively, and the working pressure was set at 0.5 Pa. The vacuum chamber was heated at 300 °C. A substrate bias voltage of −500 V was applied, and the deposition time was fixed at 4 h. All deposition parameters were fixed except the substrate rotation speed, which was varied by 0.5, 1, 1.5, 2, and 3 rpm. The detailed deposition conditions are summarized in Table 1.

### 2.2. Coatings Characterization

The surface morphology and the cross-sectional microstructure were detected by SEM field emission (JEOL JSM 7610F, JEOL Ltd., Tokyo, Japan). The chemical composition of CrAlN coatings was evaluated by EDS and WDS analysis. Roughness and surface topography were determined via atomic force microscopy (AFM-type XE Park 70, Park Systems, Gwacheon, Republic of Korea) using a tapping mode. The image scanned area was about (5 × 5) µm^2^. The friction coefficient was determined by rotative ball-on disk tests under a sliding distance of 100 m, a normal load of 5 N and a sliding speed of 3 cm/s at ambient air with a relative humidity of approximately 40% RH. A ball of alumina (Al_2_O_3_) with 6 mm diameter was used as a counter body. After the sliding test, the wear volume was investigated using a 3D optical profilometer (VEECO, Plainview, NY, USA, Wyko NT-1100). To be more precise, eight sections along the depth of the wear track were selected. The chemical composition of the wear track was determined by EDS analysis (JEOL JSM 6400F). The residual stress was calculated by the Stoney formula [28] (Equation (1)):

(1) $$\sigma =\frac{E_{S}}{6(1-\nu_{S})}\frac{e_{S}^{2}}{e_{f}}(\frac{1}{R}-\frac{1}{R_{0}})$$

where *E_S_*: Young’s modulus of the substrate, ν*_S_*: substrate Poisson’s ratio, *e_S_*: substrate thickness, *e_f_*: film thickness, *R*_0_: radius of curvature before deposition, *R*: radius of curvature after deposition. *R*_0_ and *R* were determined by 3D optical profilometer and the Gwyddion (version 2.7) software. Hardness and elastic modulus of CrAlN coatings measurement were performed by using a nanoindenter (MTS XP Nano indenter, MTS Systems Corporation, Eden Prairie, MN, USA), which is equipped with a diamond-tipped Vickers indenter using a maximum load of 750 mN. The tip radius of the indenter is about 200 µm, and its face angle is about 136°. For each sample, 12 nanoindentation tests were performed. To calculate the hardness, Rahmoun model’s was used [29]. Using this model, the indentation depth was superior to the coatings’ thickness, which permits us to calculate the hardness and the elastic modulus without the substrate effect. The adhesion strength was carried out by a micro-scratch tester (Scratch Tester Millennium 200, TRIBOtechnic, Clichy, France) with a Rockwell diamond indenter and equipped with an acoustic emission detector. To be more precise, three tests were performed for each sample.

## 3. Results

### 3.1. Chemical Composition

**Figure 2:**
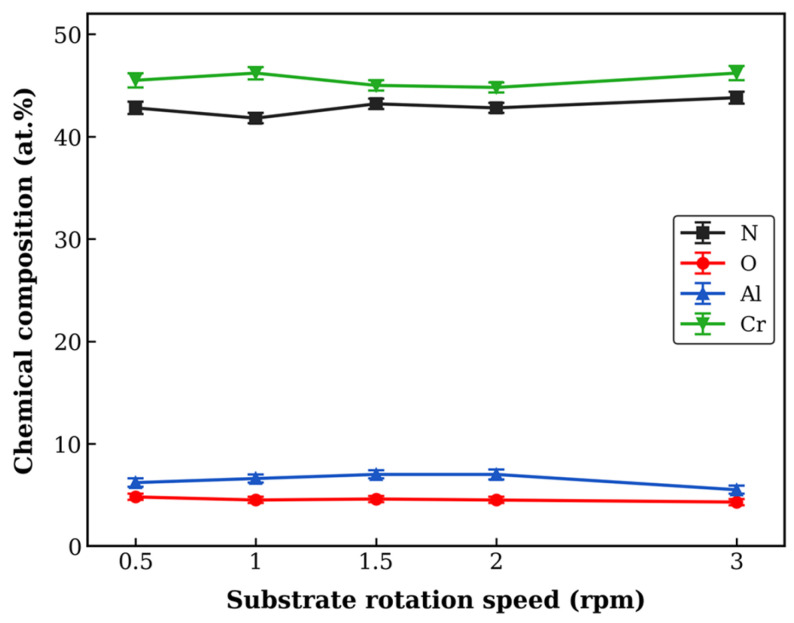
Chemical compositions of CrAlN films as a function of substrate rotation speed.

The chemical composition of CrAlN films deposited at different substrate rotation speeds is presented in Figure 2.

All films were mainly composed of Cr, Al, N, and a small amount of O. The results showed that the chemical composition of the CrAlN films remained nearly unchanged, indicating that substrate rotation speed has no influence on the chemical composition. This finding is consistent with other research [30].

**Figure 3:**
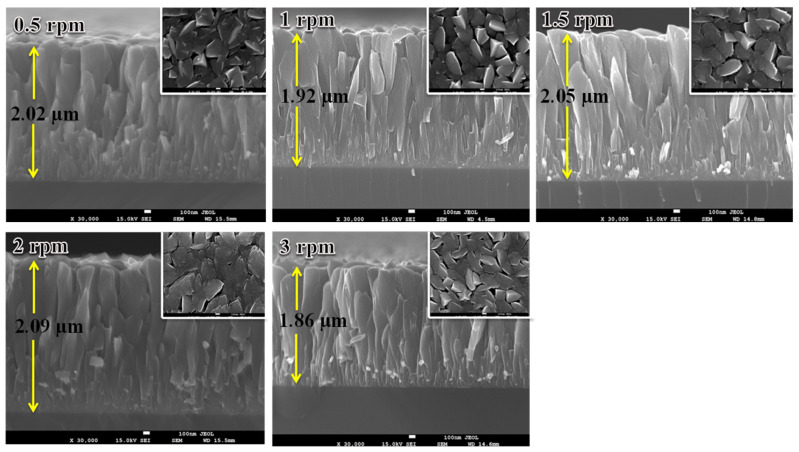
Surface and SEM cross-section observation of CrAlN coatings deposited with different rotation speeds.

Figure 3 shows the surface and SEM cross-section morphologies of CrAlN films deposited with different substrate rotation speeds.

Cross-sectional observation reveals a typical columnar structure for PVD coatings. All CrAlN films exhibit a dense and compact structure, with a preferential column orientation perpendicular to the substrate surface. The CrAlN film deposited at a rotation speed of 1.5 rpm has wider columns than the others. In contrast, the CrAlN layer deposited at 3 rpm shows the smallest column width. This denser microstructure at higher rotational speed suggests that the CrAlN grains preferentially grow vertically during the deposition process. This is very beneficial to the columnar growth pattern of coatings deposited at higher rotational speeds [31]. The thicknesses of the CrAlN coatings were 2.02 µm, 1.92 µm, 2.05 µm, 2.09 µm, and 1.86 µm at rotational speeds of 0.5, 1, 1.5, 2, and 3 rpm, respectively. No significant changes in thickness were observed with increasing rotational speed. When the substrate rotated at low speeds, its surface spent more time facing the sputtering ions (target) and remained almost perpendicular to it, promoting the formation of a denser Cr and Al structure at 0.5 and 1 rpm. As the substrate rotation speed increased, this dense structure transformed into a coarser one. This finding is consistent with earlier studies [31,32].

**Figure 4:**
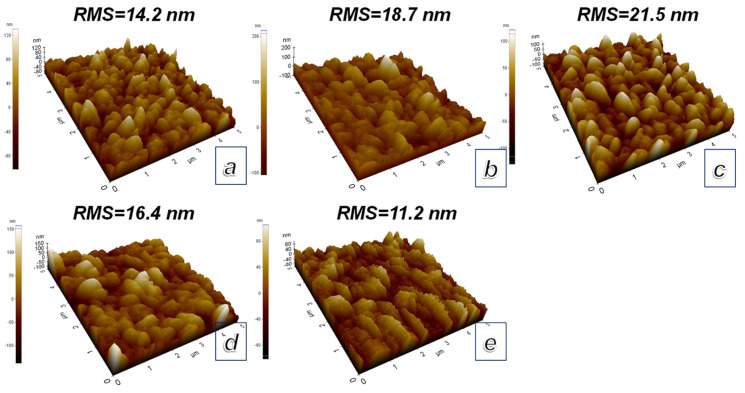
AFM images of CrAlN coatings at different substrate rotation speeds: (**a**) 0.5 rpm, (**b**) 1 rpm, (**c**) 1.5 rpm, (**d**) 2 rpm, (**e**) 3 rpm.

Surface morphology at different substrate rotation speeds is illustrated in Figure 4. All CrAlN thin films exhibited a pyramidal facet structure. Numerous gaps between grains were observed for all coatings. The surface grains of the CrAlN coating deposited at 3 rpm were smaller than those of the other samples. 

The surface morphology and roughness of CrAlN films on Si substrates were analyzed by AFM over a measured area of 5 × 5 µm^2^. Figure 4 shows typical three-dimensional AFM morphologies of CrAlN films deposited at different rotational speeds. All coatings exhibit a dense and columnar structure. The CrAlN coating obtained at a rotation speed of 1.5 rpm appears denser than the other films. As the rotational speed increases, the domes become wider. At low substrate rotational speeds, sputtering ions tend to have a high vapor incident angle (VIA), which refers to the angle between the horizontal direction of the sample substrate and the incident direction of the sputtering ions [32]. This effect, combined with the high surface injection effect and surface rebound, leads to a decrease in the effective deposition of sputtering ions on the substrate surface. As a result, good surface quality can be achieved. However, as the substrate rotational speed increases, the VIA, surface injection, and surface rebound all decrease, which causes an increase in surface roughness. At higher substrate rotational speeds, the energy of sputtering ions is reduced due to the increased distance of the substrates from the target surface. This reduction in energy is attributed to the magnetic field effect of the targets and the particle collision effect, which together decrease the energy of the sputtering ions [33]. Interestingly, this low-energy state only has an etching effect on the surface substrate. As a result, the surface roughness decreases once again. Therefore, it is possible to control the surface roughness during the sputtering process by varying the substrate’s rotational speed.

### 3.2. Mechanical Properties

**Figure 5:**
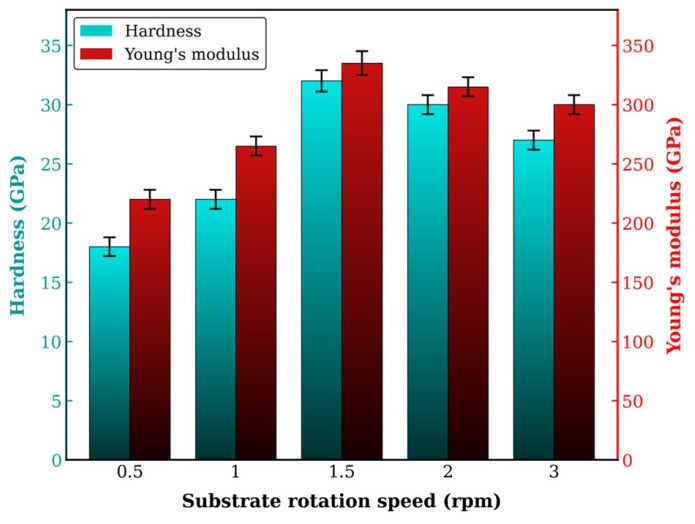
Hardness and Young’s modulus of CrAlN films obtained at various rotation speeds.

In this work, the Rahmoun and Iost model [29] was used for hardness analysis. Since this model explicitly accounts for the influence of the substrate via an area mixing law, indentation depths greater than 10% of the film thickness were used without biasing the estimation of the intrinsic hardness of the coating. Figure 5 shows the nanohardness and Young’s modulus of the as-deposited CrAlN films as a function of rotational speed.

The results show that as the rotation speed increased from 0.5 to 1.5 rpm, the hardness and Young’s modulus of CrAlN increased from 18 to 32 GPa and from 221 to 334 GPa, respectively. When the rotation speed was further increased to 3 rpm, both hardness and Young’s modulus decreased to 27 GPa and 301 GPa, respectively. The CrAlN coating deposited at a rotation speed of 0.5 rpm exhibited the lowest hardness, which may be attributed to its porous structure, as seen in Figure 3. Increasing the rotation speed improved hardness and Young’s modulus. Specifically, at a rotation speed of 1.5 rpm, the hardness reached 32 GPa and the Young’s modulus was approximately 340 GPa. When the carousel was rotated at 2 and 3 rpm, both hardness and Young’s modulus decreased slightly, reaching 28 and 304 GPa, respectively, at 3 rpm. This is verified by the results of Gu et al. [34], who produced CrWN films by DC magnetron sputtering and observed the same trends. The values found in this work are comparable to recent data published on CrAlN films produced by different PVD techniques. For instance, Kraynova et al. [35], studying HiPIMS-deposited CrAlN with varying Al/Cr ratios, reported hardness values between 26 and 29 GPa, attributing the upper range to higher Al content. Also, Bouamerène et al. [36], investigating DC magnetron CrAlN deposited at elevated temperatures, observed a maximum hardness of 28.1 GPa at 300 °C, linked to enhanced adatom mobility and densification. He et al. [27], optimizing nitrogen pressure in DC sputtering, achieved 30.6 GPa at 10 Pa. Finally, Li et al. [37], using multi-arc ion plating, reported 24.5 GPa. Several factors can affect the hardness of films, such as their stoichiometry, chemical composition, residual stresses, grain size and preferential orientation [38]. The enhancement of hardness observed when the substrate rotation speed increases from 0.5 to 1.5 rpm is primarily attributed to the improved uniformity of the plasma flux and the increased frequency of ion/species bombardment across the substrate surface. In a dual-axis planetary configuration, higher rotation frequencies reduce angular shadowing effects and promote a more homogeneous distribution of depositing species, which enhances adatom surface mobility and suppresses void nucleation. This results in a denser, less porous microstructure, a well-documented driver of hardness improvement in hard nitride coatings [32]. Additionally, more uniform exposure to reactive nitrogen and reduced preferential re-sputtering of metallic species are likely to favor a more stoichiometric nitride phase distribution, further supporting mechanical performance. Beyond 1.5 rpm, excessive flux modulation and increased re-sputtering may offset these densification benefits, leading to the observed stabilization or decrease in hardness.

By increasing substrate rotation speed from 1.5 to 3 rpm, hardness decreases. At higher rotation speeds, the bombardment of the substrate surface by ionized particles becomes too intense. This can lead to damage or disruption of the coating structure.

Mechanical properties of different CrAlN thin films deposited under different rotational speeds are presented in Table 2.

**Figure 6:**
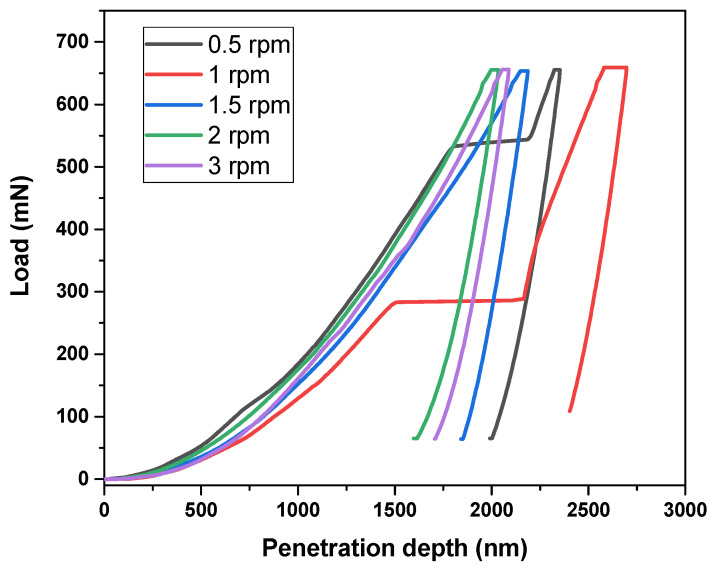
Load–unload curves of CrAlN films obtained at various rotational speeds.

To study the elastic recovery properties of CrAlN coatings, a typical nanoindentation load displacement curve is shown in Figure 6.

All CrAlN thin films represent a continuous loading–unloading process. This means that all coatings have a compact structure. The results show that CrAlN films deposited under 0.5 and 1 rpm rotation speeds exhibit pop-ins. They are manifested by a sudden increase in penetration depth without a significant increase in the applied load. This may be due to the microstructure of these two films. These pop-ins observed during loading indicate that the CrAlN film undergoes plastic deformation. These pop-ins may also be due to the rupture of the surface layer, which allows the indenter to abruptly push into the substrate. CrAlN thin films deposited under 1.5, 2 and 3 rpm substrate rotation speeds exhibit a smooth load displacement curve, indicating that the coatings are uniform. In addition, the CrAlN film deposited under 2 rpm substrate rotation speed exhibits the smallest plastic residual pressure. This indicates that the plastic deformation is the smallest and the elastic deformation is the largest.

**Figure 7:**
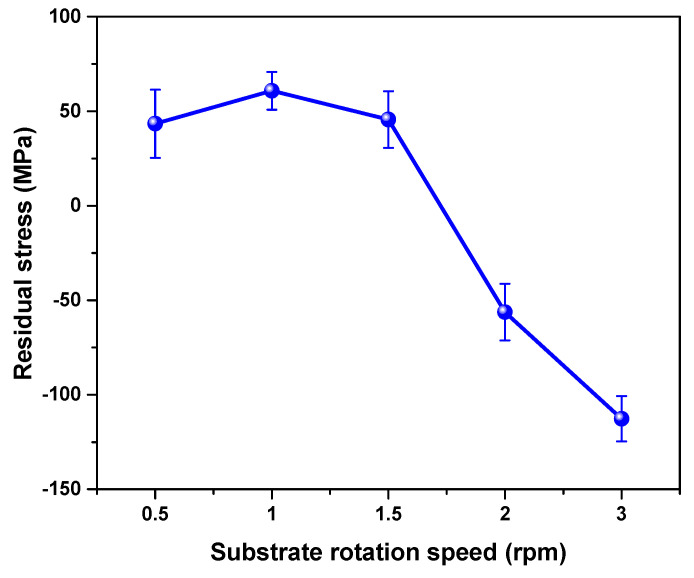
Residual stress of CrAlN coatings obtained at various rotational speeds.

Figure 7 presents the residual stress of the coatings deposited with various substrate rotation speeds.

By applying a substrate rotation speed between 0.5 and 1.5 rpm, the residual stresses are tensile and relatively stable, whereas they become compressive when the substrate rotation speed is increased to 2 and 3 rpm. According to Hoffman [39], the appearance of residual tensile stresses is related to the interaction between contiguous morphological columns. Indeed, interatomic forces act on neighboring columns, inducing elastic deformation of the grain boundaries. This deformation is compensated for the development of intra-granular tensile forces imposed by the adhesion forces between the coating and the substrate. In this case, adhesion forces are higher than the forces acting between grains. Additionally, the creation of tensile stresses can be attributed to the difference in thermal expansion coefficients between the CrAlN film and the substrate [40]. This leads to a non-homogeneous temperature distribution between the substrate and the CrAlN coating, which can make thermal stresses dominant. As a result, tensile stresses increase. Additionally, tensile stress can be attributed to the increase in ion incident energy arriving at the substrate surface, which can raise its temperature [24]. Thus, thermal stress becomes dominant. When the substrate rotation speed increases to 2 and 3 rpm, the residual stress becomes compressive. This can be attributed to a high defect density. Indeed, increasing the substrate rotation speed leads to an increase in the number of adatoms [41]. Thus, the bombardment energy increases, and the defect density also increases. This change in residual stress can also be attributed to an increase in ion energy. Indeed, upon arriving in contact with the substrate surface, atoms that etch the coating penetrate and become trapped on the substrate surface, thereby creating film growth defects. The increase in compressive stress with increasing substrate rotation speed may be ascribed to an enhanced ionization effect, which leads to the trapping of atoms deeper within the CrAlN film [40]. In general, compressive residual stresses occur during the bombardment of thin films by high-energy ions. In this case, various defects such as low-angle grain boundaries and stacking faults can arise, which may produce a deformation of the lattice parameters. Furthermore, all CrAlN coatings exhibit small residual stress values. This can be attributed to the dominance of thermal stress resulting from substrate heating. In fact, in PVD coatings, residual stress primarily arises from the combined effect of thermal stress and intrinsic stresses. In our specific case, thermal stress dominates, leading to significant stress relaxation and consequently resulting in small residual stresses.

**Figure 8:**
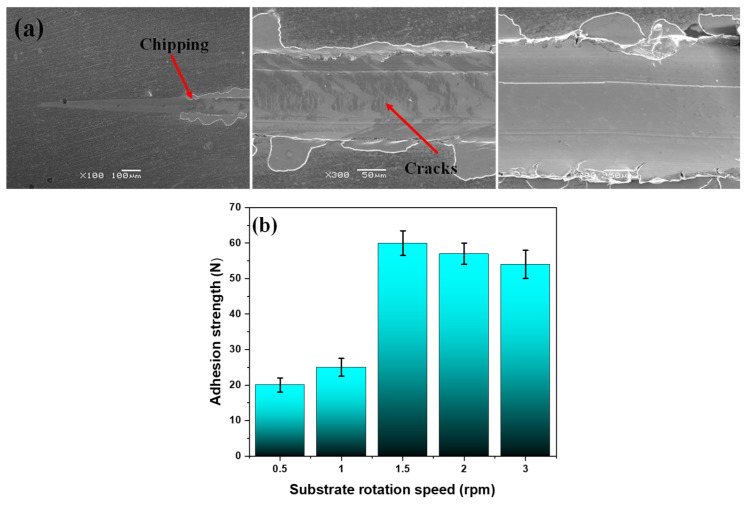
SEM image of scratch trace (**a**) (×100, ×300, ×300 respectively) and adhesion strength (**b**) of CrAlN thin films deposited with different rotational speeds.

The variation in adhesion strength obtained from scratch tests of CrAlN coatings deposited at different substrate rotation speeds is shown in Figure 8. The adhesion properties of the CrAlN films are assessed through the scratch test, and the adhesion strength reflects the resistance of the coating to spallation.

The process of coating damage following a scratch test is basically divided into three stages. At the beginning, the applied load is low, and the friction between the indenter and the coating is also low, changing only slightly. In this stage, the coating’s resistance is greater than the indenter pressure, and tiny cracks begin to appear. In the second stage, friction increases with increasing load. Cracks appear along the scratch track, and coating chipping begins. Once the load increases to a higher value, friction stabilizes, and the coating becomes completely delaminated. At this point, the indenter rubs directly against the substrate. High loads required for initial crack generation and first chipping indicate that the coating exhibits better adhesion. Figure 8a illustrates the different damage stages following a scratch test.

As clearly seen in Figure 8b, the critical load of CrAlN films is significantly improved by increasing rotational speed. Critical load values vary between 20 and 60 N. For a rotation speed of 0.5 rpm, Lc_2_ is around 20 N. As the rotational speed increases to 1.5 rpm, the adhesion strength is highly improved and reaches 60 N. Adhesion strength decreases slightly for a rotational speed of 3 rpm and reaches 54 N. We find that the critical loads have the same trend as that of the hardness (Figure 5). According to Cao et al. [26], the enhancement of critical load is related to the increase in H/E and H^3^/E^2^ values of CrAlN films due to the grain refinement. Also, microstructure can play an important role in improving the adhesion of CrAlN films. Indeed, from Figure 3, the SEM images of the surface morphology of the films obtained with rotation speeds of 0.5 and 1 rpm show nanopores. These defects can weaken the bonds between the columns and decrease the coating’s adhesion. On the other hand, the CrAlN film obtained with 1.5 rpm rotational speed has a flattened surface without nanopores, which may explain its best adhesion. The decrease in critical load adhesion when rotational speed increases from 1.5 to 3 rpm can be due to the appearance of vertical cracks, which are created because of the highly energetic ion bombardment. Then, the reduction in adhesion strength at 2 and 3 rpm rotation speeds can be ascribed to the broken texture of CrAlN films. Likewise, this reduction in adhesion strength can be explained by the decrease in compressive residual stress [42]. In fact, the increase in compressive residual stress is attributed to the accumulation defects. Consequently, the increase in compressive residual stress leads to a decrease in adhesion strength.

### 3.3. Tribological Properties

**Figure 9:**
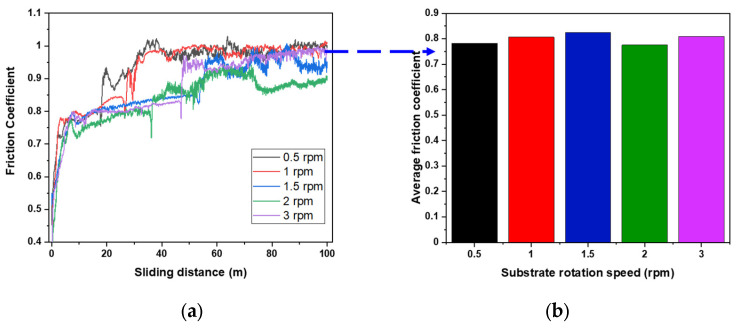
(**a**) Friction curves of CrAlN coatings deposited at different rotational speeds. (**b**) Average friction coefficient.

Figure 9 shows the friction curves of the CrAlN films as a function of rotational speed. All curves could be divided into three stages (Figure 9a). In the first stage, in the beginning, the friction coefficient of CrAlN coatings is very low, and it increases rapidly. In the second stage, the friction coefficient remains constant. In this stage, the ball is in contact with the CrAlN coatings. In the last stage, friction coefficient values increase and reach the value of the friction coefficient of the substrate. This confirms that the coating layer has been removed.

**Figure 10:**
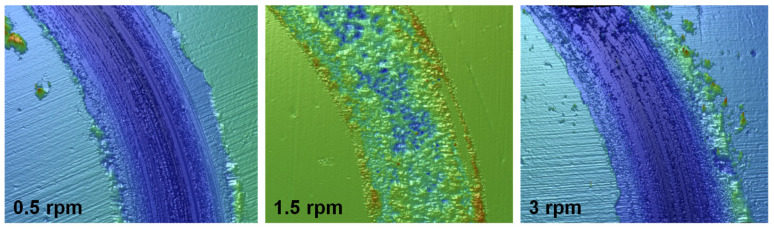
3D wear topographies of the CrAlN thin films deposited at different rotational speeds.

The results indicate that the variation in the rotational speed has no effect on the friction behavior of CrAlN films deposited with 7% at. of Al. All coatings have a friction coefficient around 0.79. This corroborates the values found by Wang et al. [43]. During the sliding of an Al_2_O_3_ ball against CrAlN films, the wear debris generated can introduce hard particles into the friction interface. This can impose the cutting effect, which can potentially damage the transfer layer. Consequently, the roughness of the friction area increases, leading to an increase in the friction coefficient. As the sliding distance increases, the impact of the cutting effect becomes more pronounced, resulting in significant changes in the roughness of friction surface [32]. We note that all CrAlN films exhibit almost the same chemical composition (Figure 2). This may be one reason why the friction coefficient does not vary with the variation in substrate rotation speed. Furthermore, in Figure 9, it is observed that the distance at which the ball interacts with the coating varies depending on the rotational speed. Specifically, at a speed of 0.5 rpm, the alumina ball interacts with the substrate after traveling 18 m. At a rotation speed of 1.5 rpm, this distance increases to 54 m, while it decreases to 47 m at a rotation speed of 3 rpm. This variation in the contact distance of the ball with the coating is likely related to the adhesion and wear resistance (as shown in Figure 8 and Figure 10). Also, this may be related to the high contact stresses, which are more important than the residual stresses at the coating/substrate interface [44].

The 3D topographies of CrAlN coatings deposited under various rotational speeds are depicted in Figure 10.

**Figure 11:**
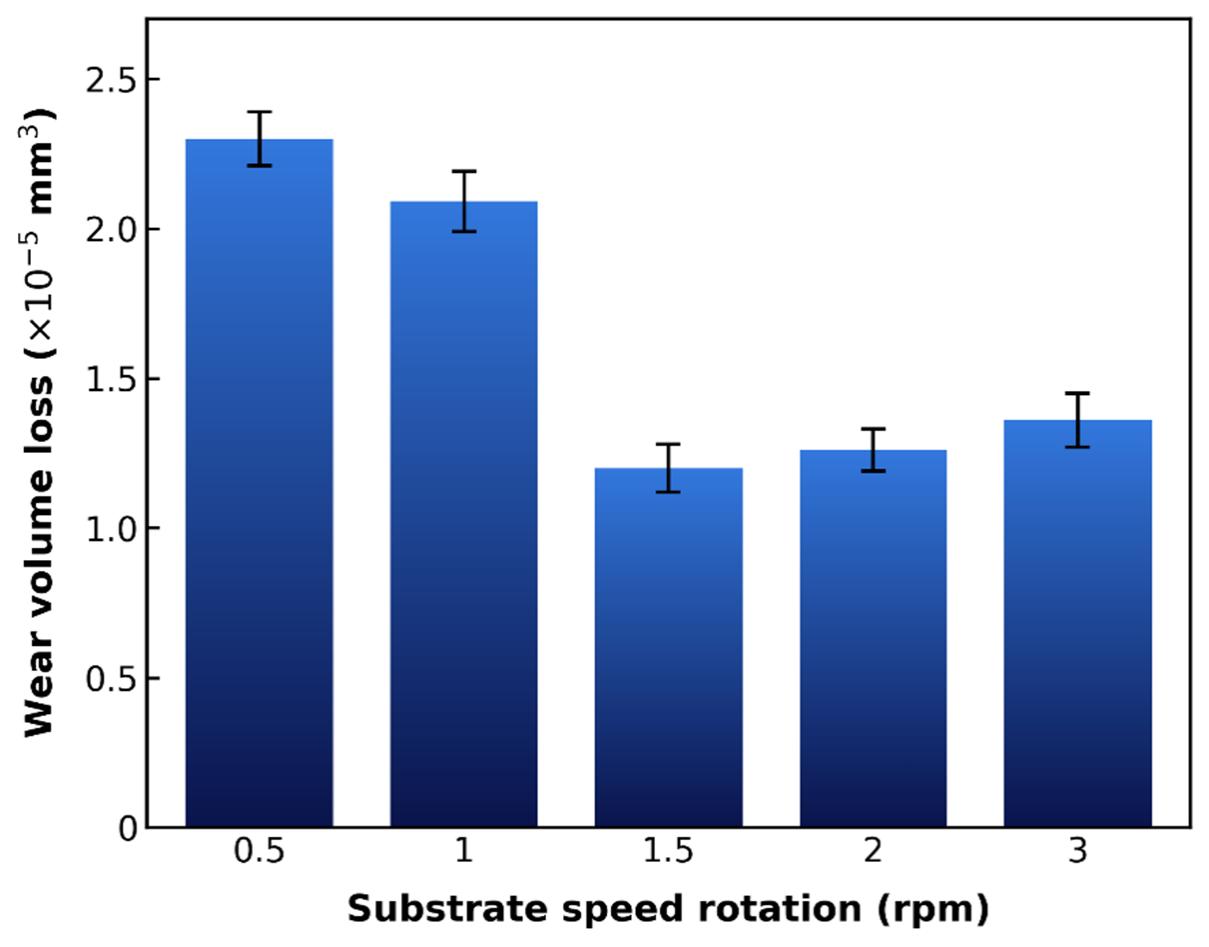
Wear volume loss of the CrAlN coatings deposited at different rotational speeds.

All wear tracks of the CrAlN coatings present obvious furrows, which indicates that the main wear mechanism of all films is abrasive wear. The results show that CrAlN film deposited under a rotational speed of 0.5 rpm exhibits the highest degree of degradation, while a coating deposited under a rotational speed of 1.5 rpm shows the least degradation. Furthermore, according to Figure 10, it can be observed that CrAlN coating deposited under the weakest rotational speed (0.5 rpm) exhibit deeper and wider wear tracks than those with higher rotational speeds. This implies that the increase in rotational speed enhances tribological properties. Overall, the outstanding interfacial properties of CrAlN coatings deposited at higher rotational speeds, as shown in Figure 7, decrease the internal stress, and also, it prevents the propagation of dislocation when the coatings are exposed to an external load and microcracks appear. Wear volume loss of CrAlN thin films deposited under different rotational speeds are visually presented in Figure 11.

The wear volume of CrAlN thin films decreases with increasing rotational speed, implying an enhancement in wear resistance. The CrAlN coating deposited at a rotation speed of 0.5 rpm displays a wear volume of 2.3 × 10^−5^ mm^3^. As the rotational speed increases, the wear volume decreases, reaching 1.22 × 10^−5^ mm^3^ at a speed of 1.5 rpm. This enhancement in wear resistance is likely ascribed to the concurrent improvement in film hardness and adhesion, as expounded in Reference [45] (refer to Figure 5 and Figure 8). Indeed, the coating deposited under a rotation speed of 1.5 rpm has the highest H^3^/E^2^ ratio. This can lead to a reduction in frictional contact pressure by distributing the applied load across a large area. Consequently, coating wear resistance will be enhanced [46]. However, a slight rate increase is observed upon increasing the rotational speed to 2 and 3 rpm. This can be elucidated by the corresponding decrease in the hardness and adhesion of the respective coatings. The wear resistance of the CrAlN thin films demonstrates competitive performance when benchmarked against the recent literature, despite variations in tribological test protocols. For comparison, Bouamerène et al. [36], testing against an Al 6061 ball over 30 m, reported wear volumes of 0.86 × 10^−3^ mm^3^ for room-temperature-deposited CrAlN and 0.51 × 10^−3^ mm^3^ for their optimal 300 °C condition. He et al. [27], using an Al_2_O_3_ counterface, 1 N load, and 24 m sliding distance, measured a wear volume of 2.51 × 10^−5^ mm^3^ for their optimal 10 Pa film. Also, Kraynova et al. [35] reported wear rates in the range of 1–13 × 10^−8^ mm^3^/Nm for HiPIMS CrAlN tested against Al_2_O_3_. While absolute wear volumes cannot be directly compared due to differences in counterface material, applied load, and sliding distance, the order of magnitude of our results at 1.5 rpm is consistent with or superior to values reported for optimized CrAlN coatings in the literature.

The superior wear resistance at 1.5 rpm is attributed to the dense, low-porosity architecture achieved at this rotation speed, which enhances the coating’s resistance to plastic deformation and microcrack initiation under contact loading. Although coatings deposited at 0.5 and 1 rpm also exhibit relatively high wear volumes, potentially facilitated by their tensile stress state, which may limit crack propagation under mild sliding, their higher morphological porosity and weaker inter-columnar cohesion facilitate localized material removal and third-body abrasion over extended sliding distances. At rotation speeds above 1.5 rpm, the accumulation of point defects and the transition to a highly compressed state reduce fracture toughness and promote subsurface crack nucleation and a slight increase in wear volume via fatigue-assisted or delamination mechanisms. Thus, the 1.5 rpm condition uniquely combines high hardness, optimal cohesion, and sufficient toughness to maximize wear resistance.

Table 3 presents a comparison of the mechanical and tribological parameters obtained in this work with those from other recent studies that deposited CrAlN coatings.

The comparative analysis presented in Table 3 highlights that the CrAlN coating deposited at the optimal rotational speed exhibits a higher hardness than that documented in recent studies, despite the diversity of deposition techniques (HiPIMS, DC sputtering, multi-arc plating). Regarding tribological behavior, the wear rate obtained (2.44 × 10^−8^ mm^3^/N·m) ranks among the lowest values in the literature, surpassing most cited references except for Kraynova et al. [35]. This combination of high hardness and low wear rate demonstrates competitive wear resistance, thus validating the effectiveness of planetary rotation as a lever for microstructural optimization. Although deposition conditions vary considerably between studies, the observed trend highlights the structuring role of rotation speed, which, by homogenizing the deposition flow and promoting morphological densification, makes it possible to achieve optimal mechanical and tribological performance without resorting to complex processes or high substrate temperatures.

**Figure 12:**
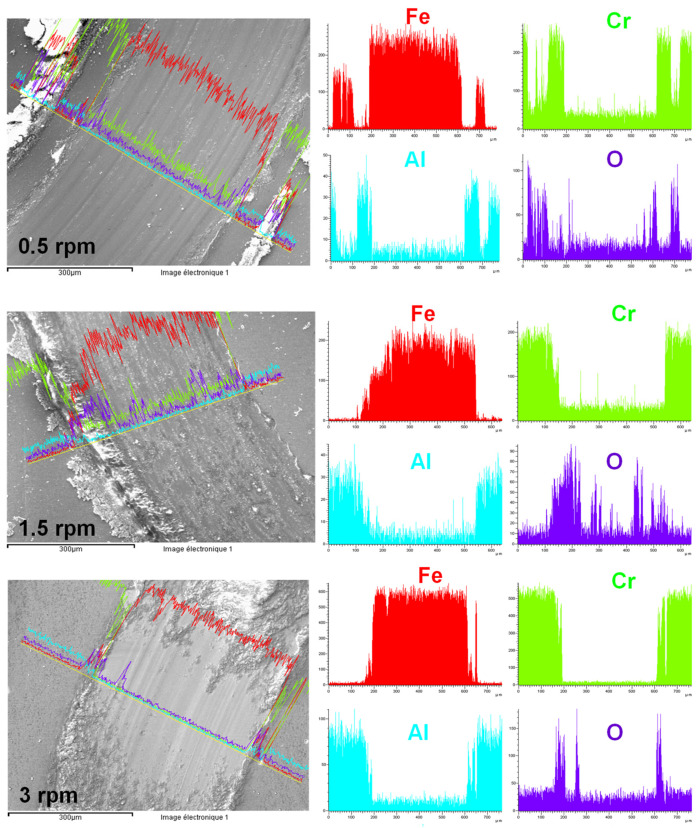
SEM images and EDS analysis of wear track of CrAlN coatings deposited under different substrate rotation speeds.

The SEM morphologies of the wear tracks of CrAlN thin films deposited at different rotational speeds and their EDS analysis are depicted in Figure 12.

Results show that the CrAlN coating deposited under a substrate rotation speed of 1.5 rpm presents the narrowest wear track, and the thin film with 0.5 substrate rotation speed showed the widest wear track. In addition, all the wear track images show a lamellar delamination. This exhibits that abrasion was the main wear mechanism. This is also very visible with profilometer images (Figure 10). SEM images coupled with EDS analysis reveal that all CrAlN thin films are completely delaminated. This is supported by the friction test results, which show that by the end of the test, all CrAlN films reached a friction coefficient of approximately 1 (Figure 9). This may be ascribed to low toughness or limited plastic deformability of these thin films. In fact, from Table 2, CrAlN coatings deposited under a substrate rotation speed of 0.5 rpm present the lowest plastic deformation. Likewise, the worn-out hard debris of CrAlN thin films acted as a third-body element with the Al_2_O_3_ ball, which led to the delamination of the CrAlN films [47]. The existence of a significant amount of oxygen, as revealed by EDS analysis, proves that tribochemical oxidation occurred during the wear test. Indeed, during dry friction, local temperature increases, and an oxide layer will be created due to the reaction between the upper layer in contact and the oxygen in the ambient atmosphere. According to Figure 12, the wear track of CrAlN coatings deposited with a substrate rotation speed of 1.5 rpm presents a small amount of Cr and Al. This means that some traces of CrAlN coatings remain on the wear track. The wear behavior of CrAlN thin films is closely linked to the improvement of mechanical properties including hardness, Young’s modulus, H/E and H^3^/E^2^ ratios. The enhancement of these properties results in an increased resistance to cracking, which is directly related to tribological behavior. This can explain the superior wear resistance of the CrAlN coating deposited with a rotation of 1.5 rpm.

## 4. Conclusions

In this work, CrAlN thin films were deposited on silicon and X50CrMoV8 by DC magnetron sputtering. The effect of substrate rotation speed on the microstructure, mechanical performance, and tribological properties was systematically investigated. The following conclusions were summarized:

The variation in substrate rotation speed influenced the column width of CrAlN thin films. However, the chemical composition did not change with the increase in rotation speed. Also, the SEM images give a columnar microstructure with slight differences in thickness.

The hardness of the CrAlN thin films is significantly influenced by the substrate rotation speed. It increases from 18 GPa at 0.5 rpm to 32 GPa at 1.5 rpm, and then slightly decreases for rotation speeds of 2 and 3 rpm. This enhancement is primarily attributed to the reduction in shadowing effects, improved uniformity of the plasma flux, and the resulting denser microstructure. The Young’s modulus and adhesion strength presented a similar trend to the hardness. However, residual stress showed a tensile stress from 0.5 to 1.5 rpm, and then, it changed to compressive stress with rotation speeds of 2 and 3 rpm.

The friction coefficient exhibits only slight changes with increasing substrate rotation speed, maintaining a value close to 0.8. Nevertheless, wear resistance is influenced by this parameter. It increased from 2.3 ×10^−5^ mm^3^ for the coating deposited under 0.5 rpm to 1.22 × 10^−5^ mm^3^ for the coating deposited under 1.5 rpm. Then, it slightly increased.

According to the results obtained, this parameter has a significant influence on the improvement of the mechanical and tribological properties of the CrAlN coating. Indeed, the deposition of this coating on the surface of wood-cutting tools allows them to increase their service life, owing to the increase in hardness and wear resistance.

While the deposition parameters clearly influence mechanical and tribological performance, the lack of direct structural characterization (XRD) prevents a definitive correlation between these properties and the crystallographic structure (e.g., grain size, phase composition). The interpretations presented here regarding densification and phase enrichment are, therefore, indirect. Future studies should incorporate structural analysis to validate these hypotheses.

## Figures and Tables

**Table 1:** Sputtering conditions for the deposition of CrAlN films.

Deposition Parameters	
Argon flow rate during etching	100 sccm
Target–substrate distance	120 mm
Argon flow rate	68.8 sccm
Nitrogen flow rate	33.3 sccm
Working gas pressure	0.5 Pa
Deposition time	4 H
Temperature	300 °C
Substrate bias voltage	−500 V
Power of target:	Cr target: 1500 W Al target: 1000 W
Substrate rotation speed	0.5; 1; 1.5; 2 and 3 rpm

**Table 2:** Mechanical properties of CrAlN thin films under different rotational speeds.

	Hardness (GPa)	Young’s Modulus (GPa)	H/E	H^3^/E^2^
0.5	18 ± 0.9	222 ± 12	0.081	0.11
1	22 ± 0.8	264 ± 11	0.083	0.15
1.5	32 ± 1.2	334 ± 12	0.095	0.29
2	30 ± 1.1	317 ± 14	0.094	0.26
3	27 ± 0.9	301 ± 10	0.089	0.21

**Table 3:** Comparison of mechanical and tribological parameters with other recent work.

Reference	Deposition Method	H (GPa)	E (GPa)	H/E	H^3^/E^2^ (GPa)	Wear Volume (mm^3^)	Wear Rate (mm^3^/N·m)	Ball
Our Work	DC Magnetron (Planetary)	32	334	0.096	0.29	1.22 × 10^−5^	2.44 × 10^−8^	Al_2_O_3_
[35]	HiPIMS	26–29	305	0.095	0.27	1.75 × 10^−3^	0.7 × 10^−8^	Al_2_O_3_
[36]	DC Magnetron	28.1	302	0.093	0.23	51.6 × 10^−3^	1.72 × 10^−6^	Al 6061
[27]	DC Magnetron	30.6	322	0.095	0.274	2.51 × 10^−5^	1.04 × 10^−6^	Al_2_O_3_
[37]	Multi-arc Ion Plating	24.5	308	0.077	0.13	9.6 × 10^−4^	3.2 × 10^−6^	Si_3_N_4_

## Data Availability

The original contributions presented in this study are included in the article. Further inquiries can be directed to the corresponding author.
